# A Novel Mutation in the Membrane Frizzled-Related Protein Gene for Posterior Microphthalmia, Non-pigmented Retinitis Pigmentosa, Optic Nerve Drusen, and Retinoschisis in a Consanguineous Family

**DOI:** 10.3389/fmed.2022.835621

**Published:** 2022-03-24

**Authors:** Xiang Ren, Yunxia Gao, Yu Lin, Xiangyu Fu, Lirong Xiao, Xiaoyue Wang, Zhibing Zeng, Li Bao, Naihong Yan, Ming Zhang, Li Tang

**Affiliations:** ^1^Ophthalmic Laboratory, Department of Ophthalmology, West China Hospital, Sichuan University, Chengdu, China; ^2^Research Laboratory of Ophthalmology and Vision Sciences, State Key Laboratory of Biotherapy, West China Hospital, Sichuan University, Chengdu, China

**Keywords:** membrane frizzled-related protein (MFRP) gene, retinoschisis, microphthalmia, retinitis pigmentosa, glaucoma

## Abstract

**Background:**

Microphthalmos (MCO) is a rare developmental defect characterized by small malformed eyes. Our study aimed to describe the clinical characteristics of posterior microphthalmos syndrome caused by a novel variant in *MFRP* gene in a Chinese patient.

**Methods:**

Complete ophthalmologic examinations were performed for the proband and proband's family members. Whole exon sequencing (WES) and Sanger sequencing were used to identify the mutated genes, and bioinformatic analysis was undertaken to predict the effect of this variant.

**Results:**

Clinical analysis showed that the proband had reduced axial length (17.95 and 17.98 mm) with normal-size corneas and shallow anterior chamber depth. Fundus photography showed scattered yellowish-white spots in the whole retina with cup-to-disc ratios of 0.95 in both eyes. Retinoschisis in the inner nuclear layer and reduced outer retina thickness were apparent on OCT examination, and optic nerve drusen demonstrated increased autofluorescence in fundus autofluorescence (FAF). Perimeter examination revealed a tubular visual field for the right eye, and electroretinography (ERG) revealed a moderately reduced rod response combined with compromised cone response. Ocular examinations of the patient's family members were unremarkable. WES revealed that the proband had homozygous mutations in c.55-1 (IVS1) G>A in intron 1 for the *MFRP* gene. Both the proband's parents and offspring were confirmed to be heterozygous by Sanger sequencing. Bioinformatic analysis showed this mutation was deleterious.

**Conclusion:**

We reported autosomal recessive posterior microphthalmia, atypical retinitis pigmentosa, and retinoschisis caused by a novel mutation in the *MFRP* gene in this consanguineous marriage family. Our study further broadens the mutation and phenotype spectrum of the *MFRP* gene in microphthalmia.

## Introduction

Microphthalmos (MCO) is a rare developmental defect characterized by small malformed eyes (small ocular globe below the expected size for chronological age, classically <2 standard deviations, or more generally, an anteroposterior diameter of <20 mm in adults) (1). Microphthalmos has many clinical phenotypes, which can occur isolated or in combination with other ocular malformations or systemic syndrome (2, 3). One third to one-half of affected individuals have microphthalmia as part of a syndrome that affects other organs and tissues in the body. These forms of the condition are described as syndromic. When microphthalmia occurs by itself, it is described as non-syndromic or isolated (4). MCO can be divided into two clinical subtypes called nanophthalmos (NNO) and posterior microphthalmia (MCOP), and nanophthalmos is classically distinguished from posterior microphthalmia based on the presence of normal corneal size and anterior chamber dimensions (5). However, the two subtypes may be associated with other eye abnormalities, including clouding of the lens of the eye (cataract), a narrowed opening of the eye (narrowed palpebral fissure), and coloboma. Additionally, affected individuals may have abnormalities called retinitis pigmentosa (RP), retinoschisis, glaucoma, and optic nerve drusen (6, 7). There were lots of discovered mutation sites for microphthalmia in Leiden Open Variation Database (LOVD) (8), ClinVar or GTR databases. Mutations in *MFRP, RPSS56, GDF3, GDF6, CHX10, MCOP1, ALDH1A3*, and *RAX* genes were reported in isolated microphthalmia (9), and isolated microphthalmia could be classified into eight subtypes according to the different mutated variants recorded in the Online Mendelian Inheritance in Man database (OMIM).

The membrane frizzled-related protein (*MFRP*) (OMIM:606227) gene has been frequently reported to cause autosomal recessive NNO or MCOP. The *MFRP* gene is located on chromosome 11q23 and encodes a transmembrane protein of 579 amino acids, which also encodes C1q and tumor necrosis factor-related protein 5 (C1QTNF5). C1QTNF5 has been identified to be associated with age-related macular degeneration (AMD) (10), retinal degeneration (11) or pigmental degeneration (12) in mice. MFRP protein is specifically strongly expressed in the medulla oblongata, hippocampus, corpus callosum and ocular tissues (mainly in retinal pigment epithelium and ciliary epithelium) (13). MFRP protein plays an important role in eye development and photoreceptor maintenance (14), explaining the risk for RP-like changes when mutated (15). *MFRP* gene mutations causing photoreceptor degeneration and RPE atrophy have been reported in mice (12). Considerable phenotypic variability was observed in microphthalmia with no clear genotype-phenotype correlations (4, 16). Moreover, the rare association of nanophthalmos/microphthalmos with pigmentary retinal dystrophy has been described in the literature in 1958 (17). Different researchers have reported the association of microphthalmos and retinitis pigmentosa (RP) or retinal degeneration with or without other ocular features which is called posterior microphthalmos pigmentary retinopathy syndrome (PMPRS). MFRP gene is one of the participant gene for PMPRS (18).

Here, we present the clinical and genetic data of a patient in which a phenotypic association of posterior microphthalmos, non-pigmented retinitis pigmentosa, retinoschisis, and optic nerve drusen was caused by a novel variant in membrane frizzled-related protein (*MFRP*). We further extended the genetic defects and phenotype of isolated microphthalmia.

## Methods

### Subjects

In this study, all the subjects in the family were identified through the proband attending the ophthalmology department at West China Hospital. This study was performed in accordance with the tenets of the Declaration of Helsinki and was approved by the Ethics Committee of West China Hospital. All individuals taking part in this research gave written informed consent. To confirm the affected status and identify whether there were any other ocular abnormalities, all the family members that participated in the study received full ophthalmic examinations, including best corrected visual acuity (BCVA), intraocular pressure (IOP), slit-lamp biomicroscopy, ultrasonography and biometry for ocular measurement (IOL-master), perimetry and fundus examination. Retinal fundus imaging was obtained by conventional 30-degree color fundus photographs, ultrawide field confocal laser scanning ophthalmoscopy (SLO), near-infrared and blue light fundus autofluorescence (FAF) imaging, and optical coherence tomography (OCT) scans. Full-field electroretinography was performed according to the International Society for Clinical Electrophysiology of Vision (ISCEV) standards 27.

### Whole Exome Sequencing (WES) and Sanger Sequence

The genomic DNA of the five individuals (designated II:1, II:2, III:1, III:2, and III:3) was isolated from peripheral blood using standard protocols using the Blood Genome Column Medium Extraction Kit (Kangweishiji, China) according to the manufacturer's instructions. The extracted DNA samples were subjected to quality control using a Qubit 2.0 fluorimeter and electrophoresis with a 0.8% agarose gel for further protocols. Whole exome library construction. Protein-coding exome enrichment was performed using xGen Exome Research Panel v2.0 (IDT, Iowa, USA), which consists of 429,826 individually synthesized and quality-controlled probes, targets a 39 Mb protein-coding region (19,396 genes) of the human genome and covers 51 Mb of end-to-end tiled probe space. Bidirectional direct Sanger sequencing was performed to validate the variant identified by next-generation sequencing. Genomic DNA was amplified by PCR using T3 Super PCR Mix, KAPA2G Robust Hotstart DNA Polymerase (2500 U) and MFRP-specific primers (Forward: TGGGACGCTGTAGCTGGCATR Reverse: CTCCTGCGGGCTTAGGGGTC, 897 bp) designed with Primer3. PCR conditions were as follows: 94°C for 5 min of initial denaturation, followed by 30 cycles of amplification of 30 s at 94°C, 30 s at 60°C, and 45 s at 72°C. High-throughput sequencing was performed by a MGISEQ-T7 series sequencer, and not <99% of the target sequences were sequenced. The sequencing process was performed by Beijing Chi gene Translational Medicine Research Center Co., Ltd, 100875, Beijing.

### Bioinformatics Analysis

Raw data were processed by fastq for adapter removal and low-quality read filtering. The paired-end reads were performed using Burrows–Wheeler Aligner (BWA) to the Ensemble GRCh37/hg19 reference genome. Base quality score recalibration together with single nucleotide polymorphism (SNP) and short indel calling was conducted using Genome Anlysis Toolkit (GATK). According to the sequence depth and variant quality, SNPs and indels were screened, and high-quality and reliable variants were obtained. The online system independently developed by Chi-gene (www.chigene.org) was used to annotate database-based minor allele frequencies (MAFs) and American College of Medical Genetics and Genomics (ACMG) practice guideline-based pathogenicity of every yielded gene variant, and the system also provided serial software packages for conservative analysis and protein product structure prediction. The databases for MAFs annotation include 1,000 genomes, dbSNP, ESP, ExAC, and Chi-gene in-house MAFs database; As a prioritized pathogenicity annotation to ACMG guidelines, the OMIM, Human Gene Mutation Database (HGMD) and ClinVar databases were used as conferences of pathogenicity of this variant. SpliceAI, TraP, and MaxEntScan were used to predict spice variants.

## Results

### Clinical Features

**Figure 1:**
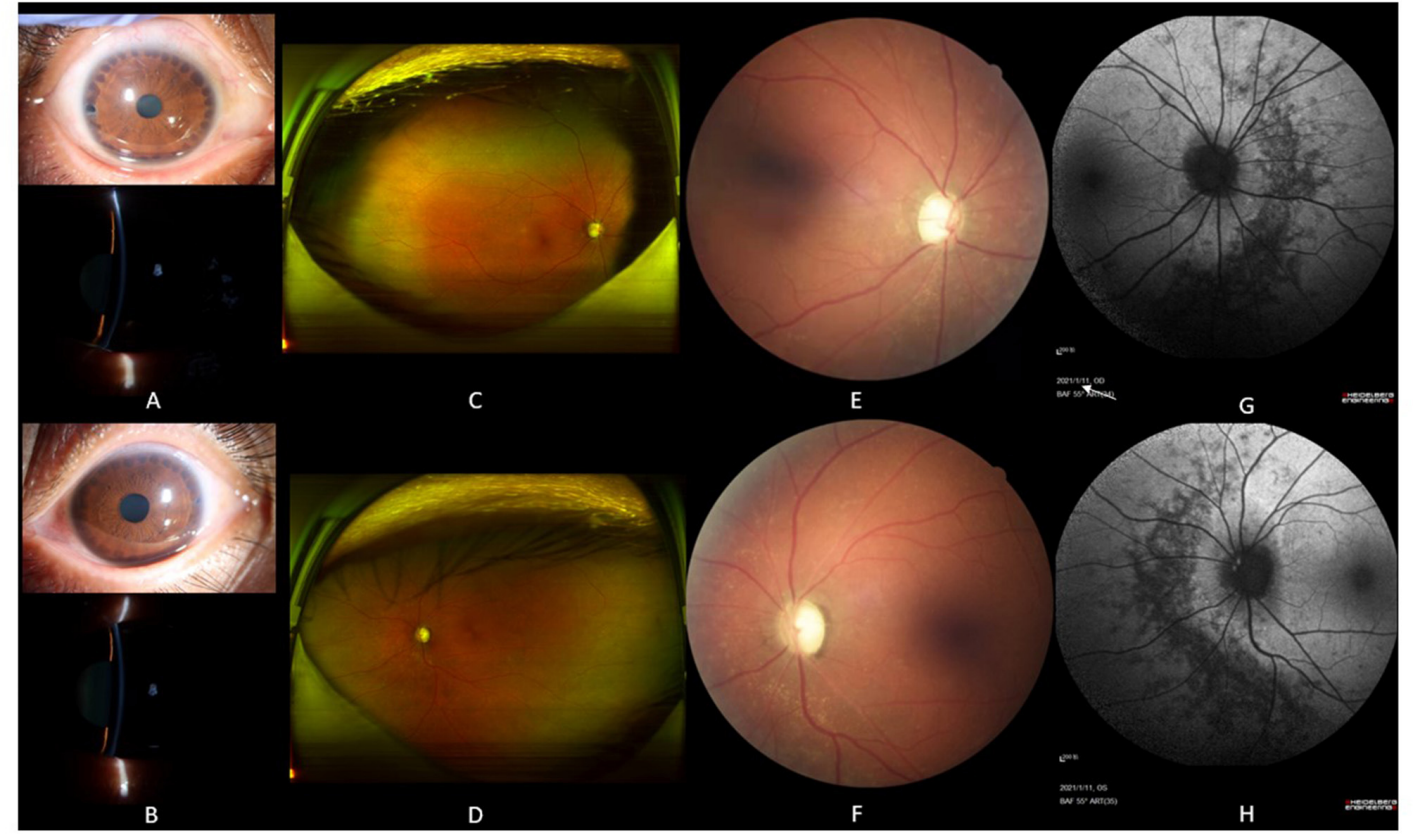
The slit lamp photos and fundoscopy for the proband. Slit lamp photos of the proband showed peripheral iris foramen after laser peripheral iridoplasty in both eyes [upper picture in **(A,B)**] and shallow anterior chamber [lower picture in **(A,B)**]. SLO presented plenty of scattered yellowish-white spots on the whole retina, and there was no bone spicule-like pigment clumping in the retina for both eyes **(C,D)**. Color fundus photography displayed enlarged cup-to-disc ratios (~0.95 for both eyes) and plenty of yellowish-white spots around the optic disc **(E,F)**. Fundus autofluorescence demonstrated decreased autofluorescence with scattered increased autofluorescence **(G,H)**. There were optic nerve drusen showed increased autofluorescence in the left eye [white arrow in **(H)**], and annular increased autofluorescence around the optic disc in the left eye **(H)**.

**Figure 2:**
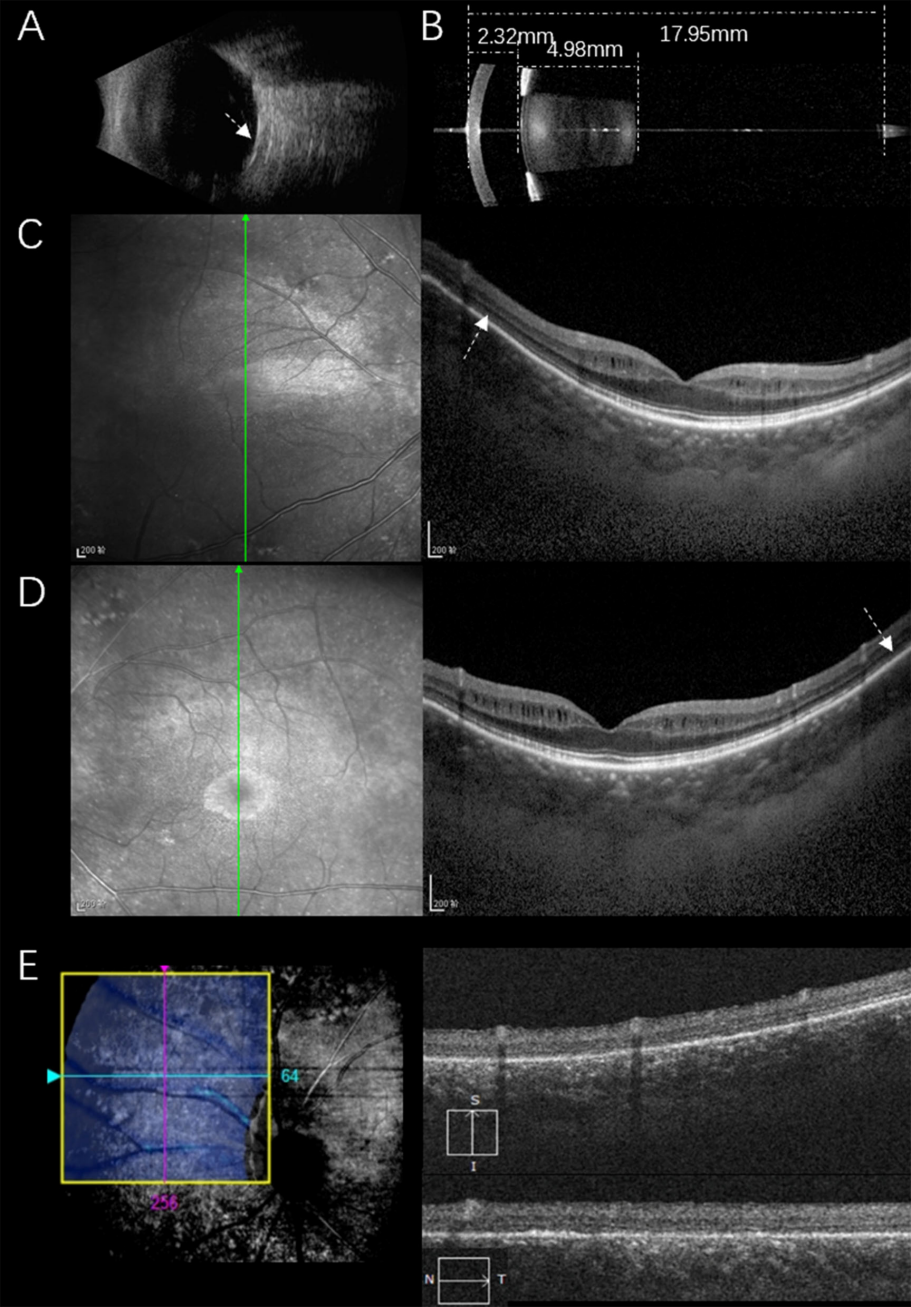
Ocular biometric measurement and OCT scan for the proband. B-mode ultrasonography showed a short eye axis, scleral thickening [white arrow in **(A)**] and IOL-Master results showed that the anterior chamber depth was 2.32 mm, the lens thickness was 4.98 mm, and the axial length was 17.95 mm **(B)**. OCT scan showed diffuse macular thickening, splitting of the inner retinal layers with discrete bridging elements at the parafovea, and the outer retinal thickness was decreased and pointed by white arrows in **(C,D)**, the reduced retinal thickness and the absent ellipsoid band were more apparent in the nasal retina around the optic disc indicating atrophy of the outer layer of the retina **(E)**.

**Figure 3:**
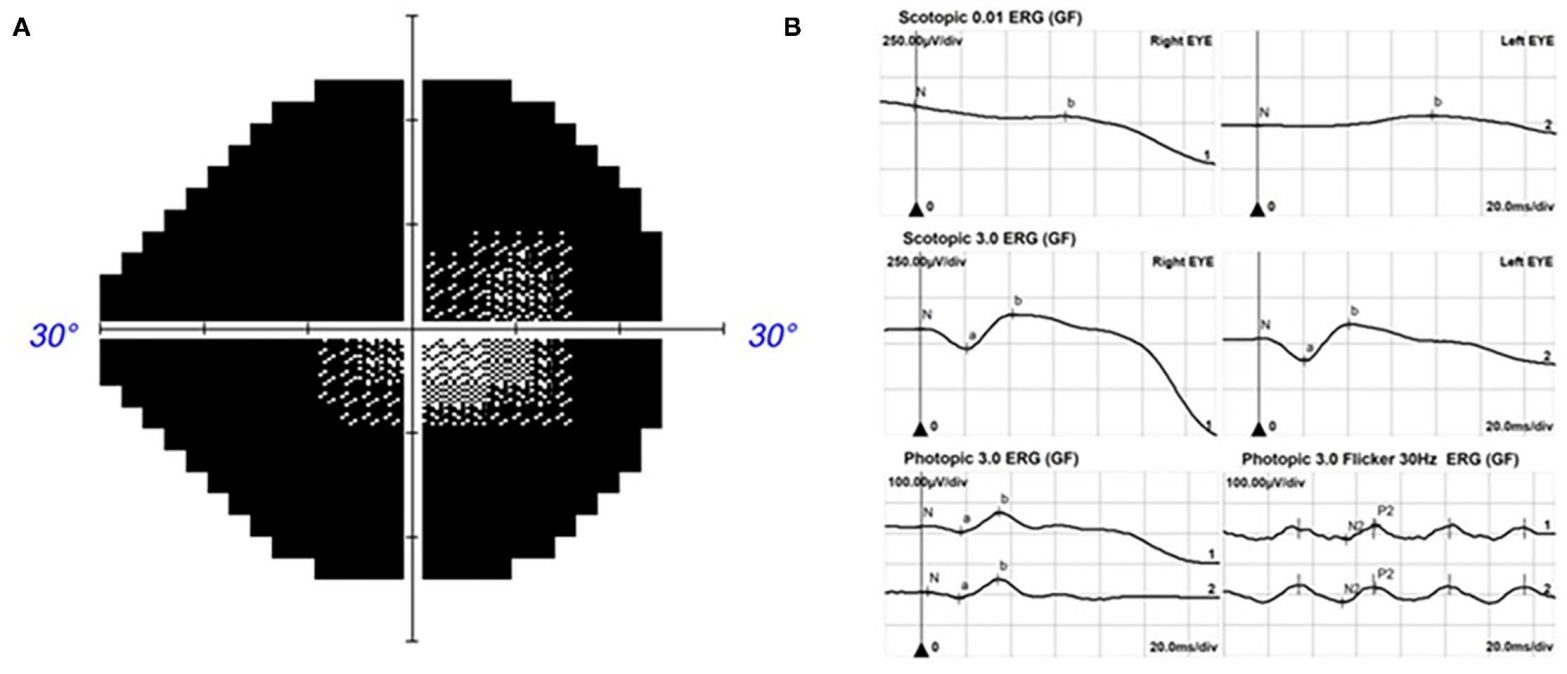
The vision field and ERG for the proband. Kinetic perimetry presented a tubular visual field in the right eye **(A)** and diminished rod responses with mild damage in cone responses on ERG **(B)**.

The proband was a 39-year-old female who was admitted to our hospital with gradual vison loss and elevated intraocular pressure (40 mmHg) and received local antiglaucoma medications followed by peripheral iridoplasty with Yttrium-Aluminum Garnet (YAG) laser and modified argon laser peripheral iridoplasty. The treatment mentioned above failed to control the IOP, so she was suggested to receive inpatient surgical treatment. Ocular examinations in the administration were as follows: the BCVA were 40/200 in the right eye, and hand movement in the left eye, IOP were 33.2 mmHg for oculus dexter (OD) and 24.2 mmHg for oculus sinister (OS). Slit-lamp biomicroscope showed the transparent cornea and slightly opacified lens with shallow anterior chamber in both eyes (Figures 1A,B). Color fundus photograph (CFP) and SLO showed enlarged cup-to-disc ratios (~0.95) (Figures 1C,D), lots of small yellowish-white spots in at the posterior pole, and peripheral retina without bone spicule-like pigment clumping in the retina for both eyes (Figures 1C–F). FAF showed symmetric decreased autofluorescence around the optic disc, and those yellowish-white spots in CFP corresponded to decreased fundus autofluorescence (Figures 1G,H), and there were optic nerve drusen (OND) showing increased autofluorescence in the left eye (Figure 1H). Ocular B-ultrasound showed reduced AL (Figure 2A), and biometric measurements of eyes (IOL-master) revealed that axial lengths were 17.95 mm (OD) and 17.98 mm (OS) (Figure 2B) with anterior chamber depth of 2.33 mm (OD) and 2.38 mm (OS), and the white-to-white distance were 11.6 mm (OD) and 12 mm (OS). OCT scan showed diffuse macular thickening resulting from retinoschisis of the inner retina in the macula and reduced retinal thickness and absent ellipsoid band (arrow in Figures 2C,D) in the outer retina of the nasal retina (Figures 2C,D), and there was no apparent deposit under the retinal pigment epithelium (RPE) on OCT. The reduced retinal thickness and the absent ellipsoid band were more apparent in the nasal retina around the optic disc indicating atrophy of the outer layer of the retina (Figure 2E). Static perimetry revealed a tubular visual field in the right eye (Figure 3A). Full-field flash-ERG showed diminished scotopic and maintained photopic activity, consistent with rod dysfunction impairment in retinitis pigmentosa (RP) (Figure 3B). These findings supported the diagnosis of atypical RP as the proband lacked typical fundoscopic signs (RPE atrophy with areas of pigment clumping and bone-spicule pigmentation). The proband had no additional somatic anomalies, intellectual disability, or hearing loss. Ocular examinations for the parents and offspring were all unremarkable.

### Bioinformatics Analysis

**Figure 4:**
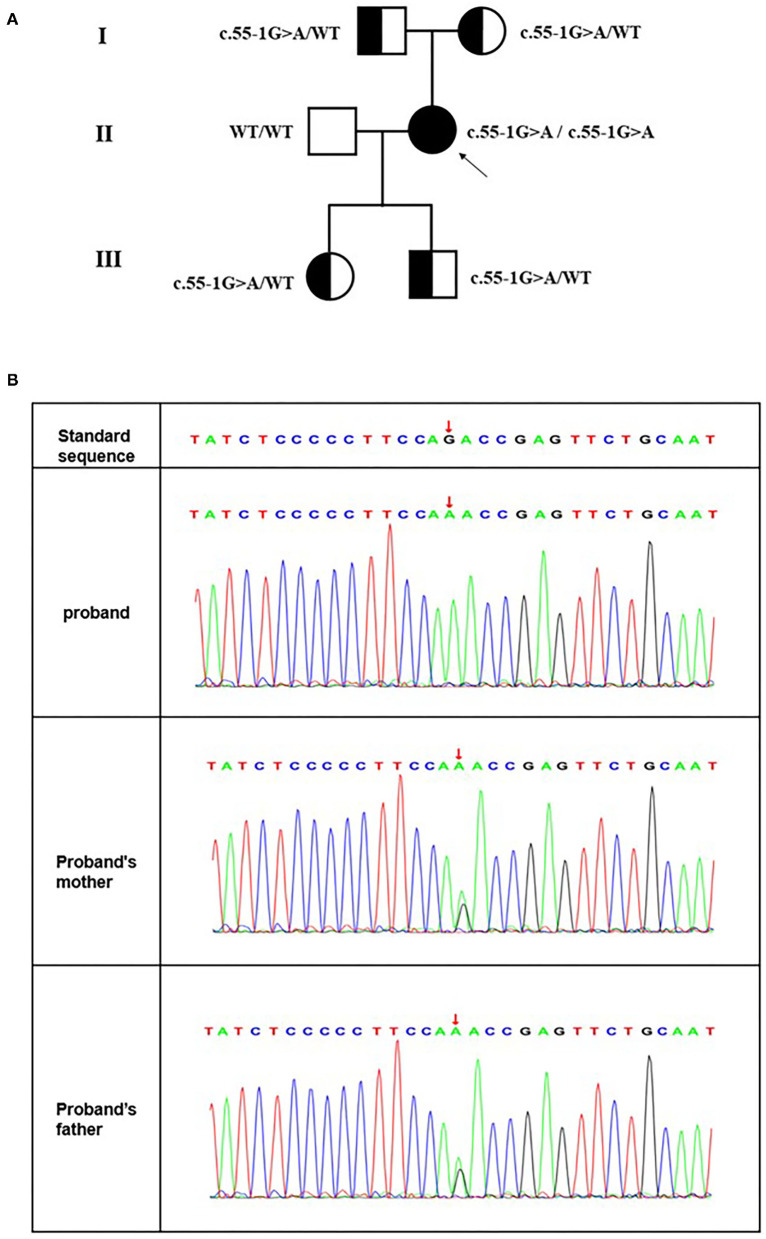
**(A)** Pedigree of the consanguineous marriage family with an autosomal recession in microphthalmos. Squares and circles represent males and females, respectively. All filled symbols and arrows indicate the proband, and half-filled symbols indicate heterozygous genotype carriers. **(B)** A novel splice site mutation [C.55-1 (IVS1) G>A] in *MRFP* for the Chinese family with congenital microphthalmia. DNA sequences of *MFRP* for the proband and her parents. The upper chromatogram of the DNA sequence from the affected proband (II: 2) shows biallelic A at the indicated position (red arrows). The lower two sequence chromatograms from the proband's parents showed both A and G.

Next-generation sequencing revealed a novel splice site variant. A splice variant was identified in intron 1 of *MRFP* (Figure 4B). No previous descriptions about it were found in ClinVar, HGMD or OMIM. The proband's parents and offspring were heterozygous carriers of one of the mutations (Figure 4B). The Transcript-ventral Pathogenicity (TraP) score is an online tool to evaluate the effect of intron single-nucleotide variants (iSNVs) and synonymous mutations on transcripts. The TraP score showed high differentiation between benign and pathogenic loci, with 99% benign loci scoring below 0.18 and all benign loci scoring below 0.37. The scores of all pathogenic loci were above 0.459. This mutation had a score of 0.463 in the TraP tool. Bioinformatic analyses confirmed the pathogenicity of the mutation as deleterious in both MaxEntScan and dbscCNV. In this case, their family members were consistent with the pathogenesis and cosegregation characteristics of autosomal recessive inheritance disease (Figure 4A). The father and mother each carrying the same heterozygous variant C.55-1 (IVS1) G>A, resulting in the homozygous variant of the same locus in the proband.

## Discussion

Congenital microphthalmia (MCO) is a heterogeneous developmental eye disease characterized by small, hyperopic eyes secondary to several etiological factors affecting the early formation of the eye (4). MCO may be isolated, complex, or syndromic based on the presence or absence of associated systemic involvement. Identifying microphthalmia as an isolated condition or as part of a syndrome has implications for counseling and can accelerate the interdisciplinary care of patients. In the present study, we described a Chinese patient suffering from isolated microphthalmia, secondary glaucoma, non-pigmented retinitis pigmentosa, optic nerve drusen, and retinoschisis. We performed whole exon sequencing to detect functional candidate genes and identified a novel splice site mutation [C.55-1 (IVS1) G>A] in membrane frizzled-related protein (MFRP) for MCO. To date, the total number of public variants in MFRP reported in the LOVD database is 108 and reported in more than 13 articles (8). Almost all of them had posterior microphthalmia, and these were originally reported as either homozygous or compound heterozygotes. In this pedigree, the clinical phenotype and genotype of the proband and their family members were consistent with the pathogenesis and cosegregation characteristics of autosomal recessive inheritance disease. The father and mother of the patient were cousins, each carrying the same heterozygous variant C.55-1 (IVS1) G>A, resulting in the homozygous variant of the same locus in their daughter (the proband). The clinical features of our patient were short axial length with normal corneal diameters and shallow anterior chamber depths. Fundus examination showed scattered yellowish-white dots in the whole retina without bone spicule-like pigment clumping in the retina for both eyes and enlarged cup-to-disc ratios. The OCT and perimetry findings suggested retinitis pigmentosa and there were optic nerve drusen on the left eye unilaterally. We predicted this variant on websites or software and defined it to be deleterious in MaxEntScan and dbscCNV.

Autosomal recessive ocular syndrome associated with microphthalmia, retinitis pigmentosa, retinoschisis, and OND has been described by different genes and mutations [*MFRP* (19), *PRSS56* (20), *MYPF* (21, 22), *CRB1, BEST1* (21), and so on]. Additional characteristics are a narrow angle between the iris and cornea, expansion of the choroidal vascular bed underlying the retinal pigment epithelium (RPE) and thickening of the scleral connective tissue surrounding the eye. Retinoschisis and OND were not always part of the phenotype in all cases (4), and some patients with *MFRP* mutation presented microphthalmia but no fundoscopic changes (23). It is well-known that papillomacular retinal folds frequently develop in subjects with nanophthalmos or microphthalmos, presumably arising from a disparity in growth between the sclera and retina and impaired vision. Macular development was unremarkable in our proband without retinal folds, although the axial length was 17.95 mm. The major risks associated with a crowded eye and a thickened sclera are uveal effusion syndrome, angle-closure glaucoma, and retinal detachment, making patients more vulnerable to intraoperative and postoperative complications. Our patient had poor vision resulting from both glaucoma and RP, and early diagnosis and timely treatment are vital for saving existing vision in microphthalmia combined with glaucoma.

In this case, the proband had no typical bone spicule-like pigment clumping in the retina and was diagnosed with RP confirmed by classic features of OCT and ERG examination. The OCT scan revealed reduced outer retinal thickness, and ERG displayed that the function of the rod and cone photoreceptor system were both compromised in our proband. FAF showed symmetric decreased autofluorescence corresponding to yellowish-white lesions. RP is a progressive disorder with a high degree of clinical heterogeneity and can be classified as the retinal degeneration disease. Yellowish-white dots associated with MFRP mutations have been reported in nanophthalmos patients, and the author referred to these lesions as retinal degeneration or retinal atrophy. Our proband had similar features of yellowish-white dots on fundus photographs and fundus autofluorescence. It has been reported that the 174delG mutation in mouse MFRP caused photoreceptor degeneration, yellowish-white dots, RPE atrophy, and activated microglia without reduced axial lengths (12). *C1QTNF5* mutation in a mouse model was reported as a human AMD animal model with the characteristics of thick deposits on the basal side of the RPE (retinal drusen), photoreceptor cell loss, and RPE thinning and atrophy. The C1QTNF5 and MFRP proteins can both be transcribed by the *MFRP* gene. MFRP protein is localized to the apical and basal RPE and ciliary region in zebrafish and mice, and mutants of MFRP showed reduced axial length, folds in the RPE, and macrophages accumulating under neurosensory tissue in zebrafish (24). These microglia/macrophages might play an important role in the pathology of retinal degeneration or RPE atrophy. As we know microglia/macrophage are involved in the pathological process of inflammation, which might explain the damage to RPE cells in this variant.

*MFRP* mutation in humans is associated with optic nerve drusen. Optic nerve drusen and retinal drusen are two different entities. Optic nerve drusen are deposits of hyaline calcific material within the head of the optic nerve and are thought to be the result of a disturbance of axoplasmic transport at the lamina cribosa, resulting in the extrusion of mitochondria filled with calcium crystals (25). Optic discs containing drusen are often described as small and crowded, lacking a physiologic cup. Our patient had an enlarged optic cup, and we found OND in FAF instead of ultrasound, as calcified OND usually displays increased echo in ultrasound. Sometimes, RP patients have OND or retinoschisis, and 4% of RP patients have OND (26). Some scholars preferred to define the OND as an independent entity from RP in microphthalmia. The mechanism of retinoschisis in microphthalmia is still not clear, and retinoschisis can be located in either the inner or outer retina as the manifestation of X-linked retinoschisis (XLRS) (27). Retinoschisis in the *MFR*P mutation can be accompanied by high reflectivity dots in OCT as well as XLRS.

In summary, we described detailed ocular abnormities of an isolated microphthalmia patient confirmed by a novel mutation in the *MFRP* gene with recessive inheritance in a consanguineous marriage family. *MFRP* mutations could be responsible for other inherited human diseases combining abnormally sized eyes and retinal degeneration. Our study also expanded the spectrum of known *MFRP* mutations and provided photographic records of patients with microphthalmia. Our paper might provide physicians and ophthalmologist with a better understanding of microphthalmia.

## Data Availability Statement

The original contributions presented in the study are publicly available. This data can be found at: NCBI, SUB10866543.

## Ethics Statement

The studies involving human participants were reviewed and approved by West China Hospital of Sichuan University. The patients/participants provided their written informed consent to participate in this study.

## Author Contributions

LT, YG, and MZ designed the study, directed the project, and interpreted the data. YG, LB, ZZ, XW, and LX performed the experiments. NY provided guidance for this project. XR and YG wrote the paper. YL, XF, NY, and MZ contributed to editing. All authors contributed to the article and approved the submitted version.

## Funding

This work was supported by the Science and Technology Innovation R&D Program of Chengdu (2021-YF05-00844-SN) and National Clinical Research Center for Geriatrics and Department of Ophthalmology, West China Hospital of Sichuan University (Z2018B17) to LT and Post-Doctor Research Project, West China Hospital, Sichuan University (2021HXBH030) to XR.

## Conflict of Interest

The authors declare that the research was conducted in the absence of any commercial or financial relationships that could be construed as a potential conflict of interest.

## Publisher's Note

All claims expressed in this article are solely those of the authors and do not necessarily represent those of their affiliated organizations, or those of the publisher, the editors and the reviewers. Any product that may be evaluated in this article, or claim that may be made by its manufacturer, is not guaranteed or endorsed by the publisher.
